# The physiological response of *Populus tremula x alba* leaves to the down-regulation of *PIP1* aquaporin gene expression under no water stress

**DOI:** 10.3389/fpls.2013.00507

**Published:** 2013-12-13

**Authors:** Francesca Secchi, Maciej A. Zwieniecki

**Affiliations:** Department of Plant Sciences, University of California DavisDavis, CA, USA

**Keywords:** transgenic poplar, aquaporin, leaf hydraulic resistance, mesophyll conductance, photosynthesis

## Abstract

In order to study the role of *PIP1* aquaporins in leaf water and CO_2_ transport, several lines of *PIP1*-deficient transgenic *Populus tremula x alba* were generated using a reverse genetic approach. These transgenic lines displayed no visible developmental or morphological phenotypes when grown under conditions of no water stress. Major photosynthetic parameters were also not affected by *PIP1* down regulation. However, low levels of *PIP1* expression resulted in greater leaf hydraulic resistance (an increase of 27%), which effectively implicated *PIP1* role in water transport. Additionally, the expression level of *PIP1* genes in the various transgenic lines was correlated with reductions in mesophyll conductance to CO_2_ (g_m_), suggesting that in poplar, these aquaporins influenced membrane permeability to CO_2_. Overall, although analysis showed that *PIP1* genes contributed to the mass transfer of water and CO_2_ in poplar leaves, their down-regulation did not dramatically impair the physiological needs of this fast growing tree when cultivated under conditions of no stress.

## Introduction

Water use efficiency (WUE) describes one of the major plant tradeoffs between rates of photosynthesis and transpiration (Gilbert et al., 2011). In general, increases in transpiration rates lead to higher photosynthetic activity. However, leaf hydraulic resistance often determines leaf transpiration capacity and is thus, directly related to carbon dioxide uptake (Brodribb et al., 2005). What is even more important, both water and CO_2_ share important parts of their transport paths in leaves, including not only the gas phase but also the liquid phase where their movement through cellular membranes is facilitated by water channels (aquaporins) (Heinen et al., 2009). The investment in the abundance of water channels would provide two major benefits. (1) It would facilitate water fluxes across the plant by reducing hydraulic resistance for water uptake in roots and improving water distribution in leaves allowing for the maintenance of relatively high stomatal conductance (g*_s_*) and so facilitating CO_2_ diffusion into leaves. (2) Investment in aquaporins would increase mesophyll conductance (g_m_) facilitating passage of CO_2_ from air to chloroplasts. Thus, improving our understanding of the role of aquaporins in leaves remains an important topic of research agenda.

The plasma membrane proteins (PIP) family is divided into two subfamilies: PIP2 and PIP1. PIP2 proteins have been shown to be major channels for water (Chrispeels et al., 2001), PIP1 proteins have been shown to facilitate both, the diffusion of CO_2_ (Uehlein et al., 2003) and supplement water permeability in conjunction with PIP2 proteins (Fetter et al., 2004; Temmei et al., 2005). Analysis of aquaporin contribution to membrane permeability to water or CO_2_ usually comes from studies aiming at blocking aquaporins using heavy metals (Barone et al., 1997; Hukin et al., 2002; Niemietz and Tyerman, 2002), ATP (Alves et al., 2001; Martinez-Ballesta et al., 2003) or anoxia (Tournaire-Roux et al., 2003; Frick et al., 2013). Most of these studies are related to analysis of water uptake by roots as infiltration of leaves with chemical compounds is much more difficult to achieve. However, a few reports showed that leaves of *Vicia faba* and *Phaseolus vulgaris* had limited mesophyll CO_2_ conductance when treated with mercury chloride, suggesting an involvement of aquaporins in CO_2_ diffusion across plasma membranes (Terashima and Ono, 2002). The problems of using chemical treatments to block aquaporins in leaves can be overcome with the use of transgenic lines with down or up-regulated expression of genes from the aquaporin's family (Hanba et al., 2004; Heckwolf et al., 2011; Uehlein et al., 2012). The majority of information we have up to date comes from analysis of the AQP1 water channel isolated from tobacco (Siefritz et al., 2001; Flexas et al., 2006) and PIP1;2 from *Arabidopsis thaliana* (Heckwolf et al., 2011). It was shown through functional assay and *in vivo* studies that NtAQP1 was associated with facilitating the diffusion of CO_2_ as well as water (Siefritz et al., 2001; Uehlein et al., 2003, 2008; Flexas et al., 2006) and AtPIP1;2 was a relevant facilitator of CO_2_ diffusion (Heckwolf et al., 2011; Uehlein et al., 2012). As both genes are members of the PIP1 subfamily, one can expect that a reduction in the expression level of *PIP1* genes should influence both photosynthesis via a reduction in g_m_, and water transport via an increase of leaf hydraulic resistance. If the role of PIP1s is truly important then a reduction in the level of their gene expression should result in detrimental effects related to photosynthetic parameters and growth especially in productive species like poplar.

Species from the genus *Populus* are generally fast growing, short lived trees with high photosynthetic and transpiration rates making them a valuable species for biofuel production, wood for pulp, and residential areas that are in need of fast shade (Isebrands and Richardson, 2013). These physiological characters are necessary from the perspective of natural history of the genus as it evolved to often be one of the first trees in succession series on land opened following natural disturbance, where competitive success depends on fast growth rates but not necessarily on efficiencies of resource use. In nature, most of the species from that genus grow in relatively mesic habitats with easy access to water and nutrients (Isebrands and Richardson, 2013). High availability of light, water, and nutrients in naturally disturbed areas (fire, hurricane etc.) is only beneficial to species that can capture the resource faster than their competitors. This strategy does not promote efficiency in resource utilization but relies on a maximization of physiological traits that result in fast growth. When compared with other tree species *Populus* might look almost wasteful with its low WUE (Brueck, 2008) and relatively high nitrogen content (Niinemets, 1997). Thus, fast growing hybrid poplar might be a good subject to determine the role of *PIP1* aquaporins in the physiology of both CO_2_ uptake and leaf hydraulics. We used transgenic plants with down-regulated expression of *PIP1* genes, with an expectation that performance of this fast growing species would be significantly impaired by a reduction in its capacity to transport water and CO_2_ across membranes.

## Results

### Populus transformation

**Figure 1:**
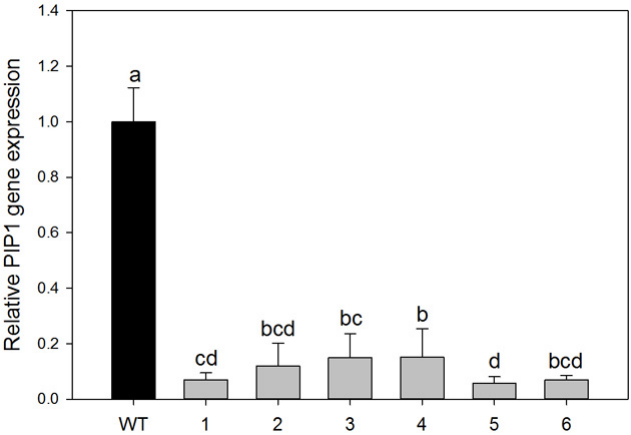
**Relative expression of *PIP1* genes in the leaves of wild-type and transgenic lines**. Each histogram is the average of three independent biological samples with two technical replicates; error bars represent SE. Letters denote homogeneous groups based on the Fisher LSD method corrected for the multiple comparisons. There is a significant difference between relative gene expression in the seven different plant groups (wt and the six transgenic lines, 1–6), (*P* < 0.001, One Way ANOVA test).

Down-regulation of *PIP1* gene expression in hybrid poplar (*Populus tremula x alba*) was achieved by using a ~270-bp hairpin RNAi construct designed from sequences in the poplar *PIP1* subfamily. Primers were specifically designed for alignment on PIP1 subfamily conserved domains (on five PIP1 isoforms from *P. trichocarpa*). Agrobacterium-mediated transformation yielded numerous independent transformants. We analyzed 22 propagated lines and PCR revealed that all contained the transgene product of the expected size for the kanamycin resistance marker *nptII* gene. From those, six different transformed lines were randomly chosen for molecular and physiological analyses. To estimate levels of *PIP1* subfamily down-regulation in the resulting transgenic plants the qRT-PCR analyses were performed on leaves of mature plants. The target of the RNAi construct clearly showed significant down-regulation in all of the tested transgenic lines. *PIP1* transcription levels were reduced by 93–85% among lines (Figure 1; ANOVA *p* < 0.001). Transgenic line 5 was characterized by lower expression levels of PIP1 genes and was also significantly different from lines 3 and 4.

**Figure 2:**
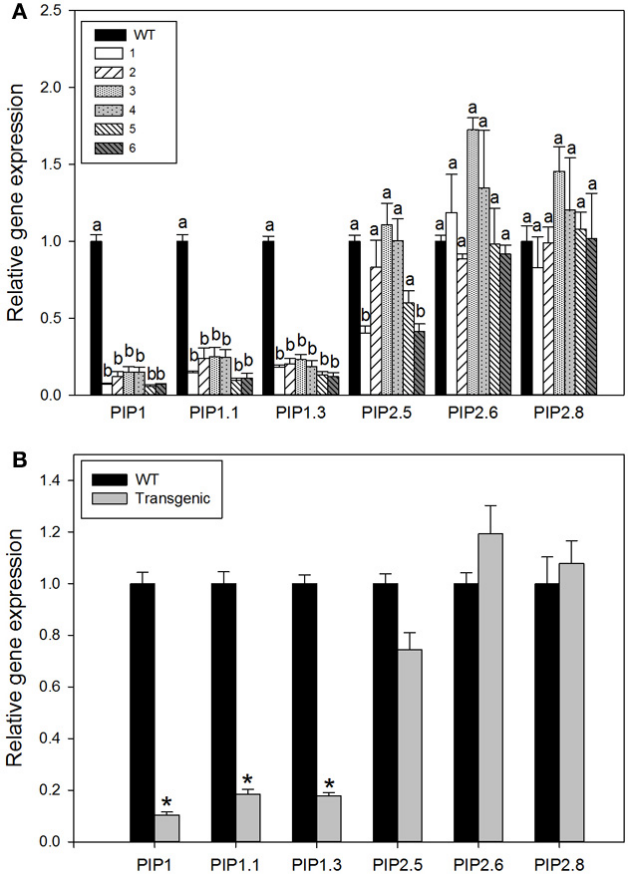
**Relative expression of aquaporin genes in the leaves of wild-type and transgenic lines**. Each histogram is the average of three independent biological samples with two technical replicates; the error bars represent SE. **(A)** Relative expression of single gene for each plant group is shown. Letters denote homogeneous groups based on the Fisher LSD corrected for the multiple comparisons. ANOVA analysis revealed presence of significant differences between wild-type and transgenic lines for all *PIP1* genes tested and for PIP2.5 gene (*p* < 0.05). **(B)** Relative gene expression for pooled data from all transgenic lines tested against expression level in wild-type plants. Stars denote significant difference (*P* < 0.001, Student's *t*-test).

In order to confirm the down regulation of different isoforms belonging to the same subfamily the expression level of two isoforms PIP1.1 and PIP1.3 were monitored. These genes were chosen because they were the most highly expressed genes in *P. trichocarpa* leaf tissue (Secchi et al., 2009). Both genes were strongly down-regulated in all transgenic lines (Figure 2A); however, we did not measured their protein abundance and thus, strong down gene regulation could be not coincided to the same strong protein suppression, although there are evidences were reduced abundance of protein was consistent with the previous analysis of RNA expression (Flexas et al., 2006).

To compare the effect of our construct on expression of the closely related PIP2 subfamily, we measured the expression of three different members; PIP2.5, PIP2.6, and PIP2.8. In general, their transcript levels were not significantly different among lines. The expression was in some cases higher and some cases lower than in wild-type plants (Figure 2A). Thus, no compensatory responses occurred in members of PIP2 gene subfamily in response to *PIP1* down-regulation (Figure 2B).

### Effect of down-regulation of PIP1 expression on leaf gas exchange

Transgenic lines and wild-type plants were grown under the same conditions in the greenhouse (for details see methods). They were phenotypically undistinguishable. No differences were found in the specific leaf area (SLA) and chlorophyll content (Table 1). In addition, the maturation process of the leaves, estimated from the changes in chlorophyll content in growing leaves, was not different among lines (Supplemental Figure 1). There were no statistical differences in basic photosynthetic parameters (net CO_2_ assimilation [A_N_], stomatal conductance to water [g_s_], RuBP regeneration rate [J], maximum rate of Rubisco carboxylation [Vc_max_], or mitochondrial respiration in the light [R_d_]; Table 1).

**Table 1:** **Photosynthetic parameters, physiological characteristics and isotope analysis of the studied genotypes**.

	**WT**	**1**	**2**	**3**	**4**	**5**	**6**	***p-value***
A_max_ (μmol CO_2_ m^‒2^ s^‒1^)	16.24, 0.52	17.75, 2.19	18.33, 0.82	18.19, 1.19	19.61, 1.17	20.39, 0.98	15.35, 1.34	0.098
A_N_ (μmol CO_2_ m^‒2^ s^‒1^)	13.17, 0.49	14.23, 1.32	13.66, 1.16	14.50, 0.82	15.08, 0.59	15.07, 0.89	11.95, 0.98	0.23
g_s_ max (mol m^‒2^ s^‒1^)	0.292, 0.007	0.329, 0.020	0.313, 0.033	0.296, 0.013	0.291, 0.012	0.300, 0.019	0.316, 0.009	0.517
g_s_ (mol m^‒2^ s^‒1^)	0.183, 0.017	0.269, 0.028	0.207, 0.061	0.211, 0.016	0.198, 0.017	0.205, 0.038	0.168, 0.038	0.316
g_m_ (μmol CO_2_ m^‒2^ s^‒1^ Pa^‒1^)	13.90 ± 1.15	4.97^*^ ± 0.54	4.59^*^ ± 1.05	7.42^*^ ± 0.84	6.15^*^ ± 0.58	4.33^*^ ± 0.54	3.47^*^ ± 0.63	<0.001
J (μmol m^‒2^ s^‒1^)	86.75, 2.04	92.20, 9.76	94, 9.77	97.25, 6.61	102.80, 5.27	111.60^*^ ± 5.05	82.00, 1.18	0.02
V_c_ max (μmol m^‒2^ s^‒1^)	62.75, 2.20	68.20, 8.99	71.33, 3.84	71.75, 7.05	78.00, 4.64	89.00^*^ ± 4.99	62.33, 1.86	0.016
R_d_ (μmol m^‒2^ s^‒1^)	1.13, 2.20	1.22, 0.10	1.16, 0.17	1.48, 0.09	1.14, 0.12	1.38, 0.13	1.13, 0.34	0.405
Chlorophyll (SPAD values)	41.40, 1.62	37.42, 2.45	39.30, 0.58	39.93, 2.23	40.06, 1.17	41.84, 2.18	35.47, 4.21	0.451
SLA (m^2^ g^‒1^)	0.0192, 0.001	0.0198, 0.002	0.0198, 0.002	0.0202, 0.001	0.0196, 0.001	0.0184, 0.001	0.0202, 0.003	0.977
δ 13C	‒30.52, 0.43	‒31.27, 0.78	‒30.81, 0.72	‒30.39, 0.46	‒30.62, 0.55	‒30.02, 0.44	‒30.34, 0.07	0.758
C Amount (μg mg^‒1^)	460.98, 4.45	456.95, 3.78	470.10, 10.64	468.61, 4.02	467.93, 1.59	463.93, 3.72	464.00, 5.78	0.475
δ 15N	5.84, 0.13	6.05, 0.42	6.45, 0.17	6.55^*^ ± 0.10	6.24, 0.14	5.88, 0.10	6.35, 0.15	0.227
N Amount (μg mg^‒1^)	30.93, 1.81	37.90, 2.77	41.26, 5.94	40.23, 1.88	39.83, 2.14	36.07, 3.50	32.59, 4.71	0.206

Values are means ± SE.

* Statistically significant differences from post-hoc Fisher least significant difference (LSD with α =0.05) corrected for the multiple comparisons. P-value represents result of test for significant differences from ANOVA analysis. Amax, maximum photosynthesis; AN, net photosynthesis; gmax, maximum stomatal conductance; gs, stomatal conductance; gm, mesophyll conductance; J, electron transport rate; Vc max, maximum rate of Rubisco carboxylation; Rd, mitochondrial respiration in the light; SLA, specific leaf area; δ13C, isotopic composition of carbon; C, carbon; δ 15N, isotopic composition of nitrogen; N, nitrogen.

**Figure 3:**
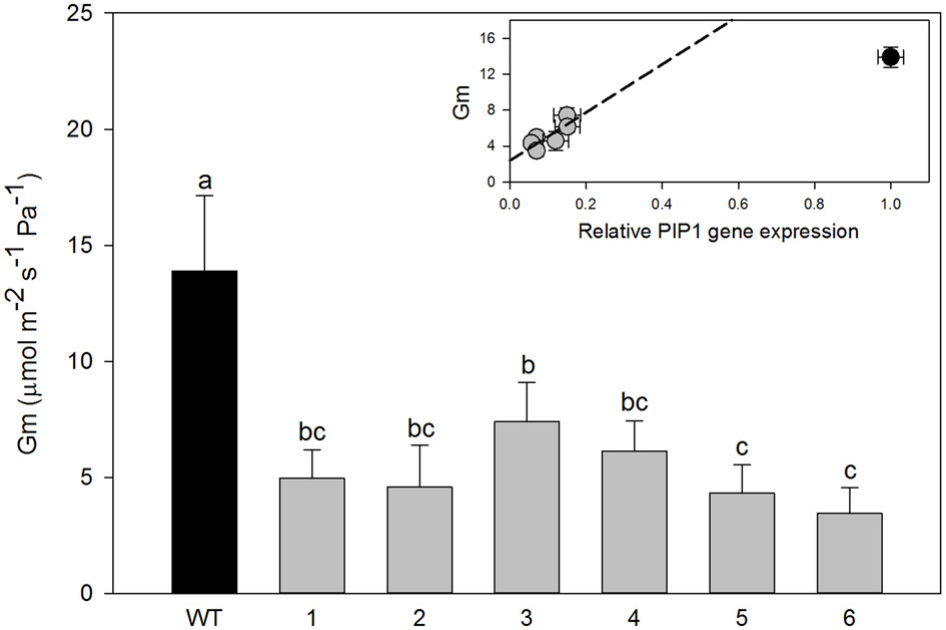
**Mesophyll conductance to CO_2_ (g_m_) in wild-type and transgenic lines**. Data are means of g_m_ for all available plants from transgenic lines (*n* = 3–8); the error bars represent SD corrected for the multiple comparisons. One Way ANOVA test suggests significant differences between wild-type and transgenic lines (*P* < 0.001). Letters denote homogeneous groups based on Fisher LSD method. Inset figure; g_m_ values of lines were plotted for PIP1 relative expression. Bars denote SE. Black circle, wild-type plants; gray circles, transgenic lines.

**Figure 4:**
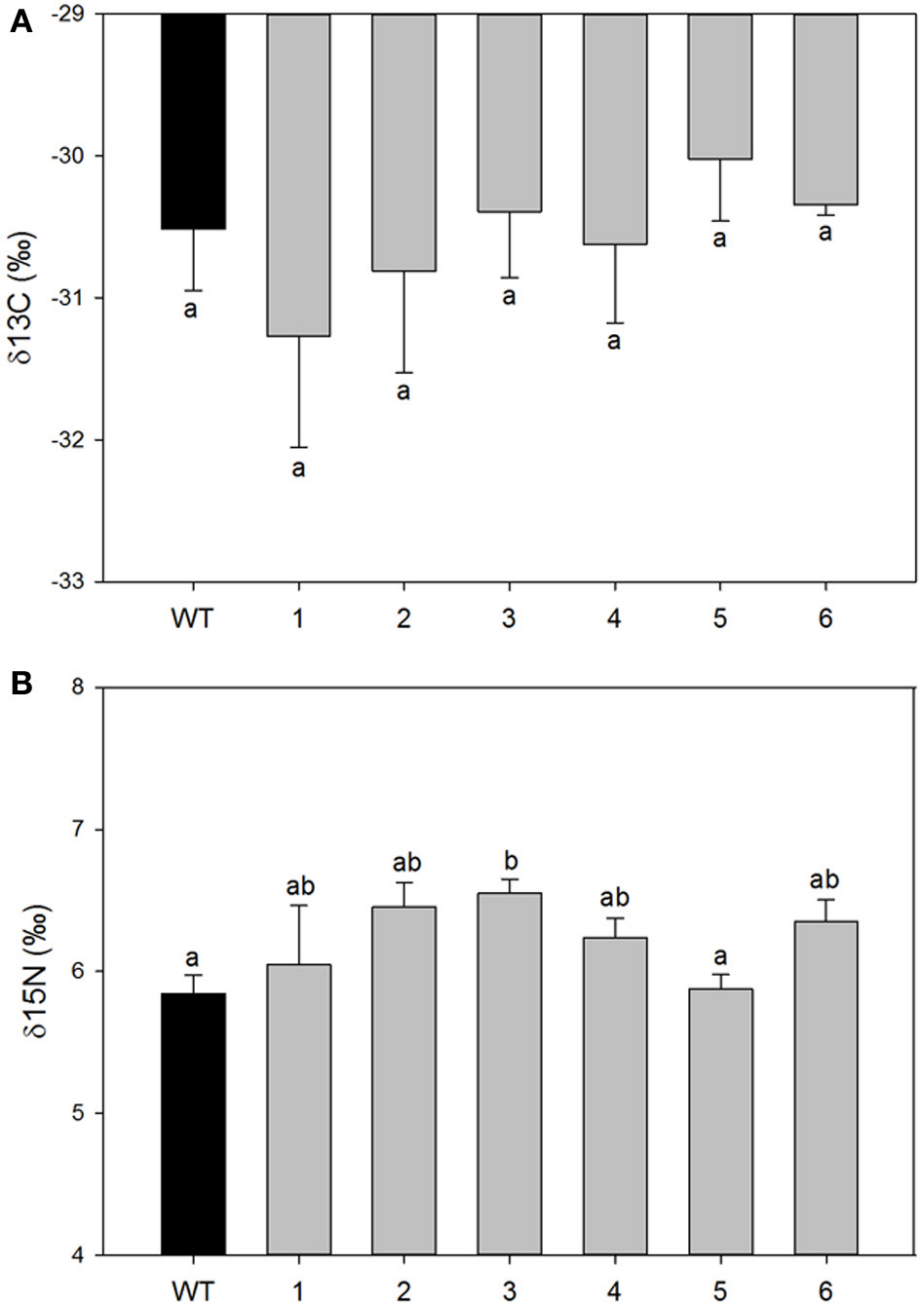
**Isotopic composition of carbon (A) expressed as δ^13^C and nitrogen (B) expressed as δ^15^N in leaves collected from wild-type and transgenic lines**. Each histogram is the average of all available plants from transgenic lines (*n* = 3–8); the error bars represent SE. There were no differences among control and transformed plants in δ^13^C signature. Letters denote homogeneous groups based on Fisher LSD method.

However, significant differences were found in mesophyll conductance to CO_2_ (g_m_) estimated using CO_2_ curve-fitting method in combination with chlorophyll fluorescence to better estimate limitation states. As estimation of g_m_ using this method might be influenced by curve parameterization, we provide raw data for each plant such that reader can build their own feel for the validity of the conclusion (SOM). In four different transgenic lines (1, 2, 5, and 6), g_m_ was less than half of that determined for wild-type plants. In the two remaining lines (3 and 4) there was a statistically significant reduction of g_m_ compared with the wild-type plants although it was physiologically less pronounced (Figure 3). Estimated mesophyll conductance (g_m_) was positively correlated with the level of down-regulation of *PIP1* expression (*R*^2^ = 0.655, *p* = 0.05 and *df* = 1,4) even when analyzed only for the transgenic lines. This suggests that the relation between *PIP1* down regulation and the observed reduction of mesophyll conductance to CO_2_ might be causative and that estimation of the g_m_ using CO_2_ curve fitting might reflect a real trend. An analysis of carbon isotopic composition (δ^13^C) showed no significant differences between wild-type and transgenic lines (Figure 4A, ANOVA *p* = 0.74 or Kruskal–Wallis ANOVA non-parametric test *p* = 0.36) decreasing the physiological importance of the observed g_m_ reduction. A non-parametric analysis showed significant increases of nitrogen isotopic composition (δ^15^N) in transgenic plants (Figure 4B, Kruskal–Wallis ANOVA non-parametric test *p* = 0.023) although this increase was very small and not picked up by regular analysis of variance (*p* = 0.23). *Post-hoc* LSD test showed that only transgenic line 3 had a significant increase in δ^15^N.

### Effect of down-regulation of PIP1 expression on leaf rehydration kinetics

**Figure 5:**
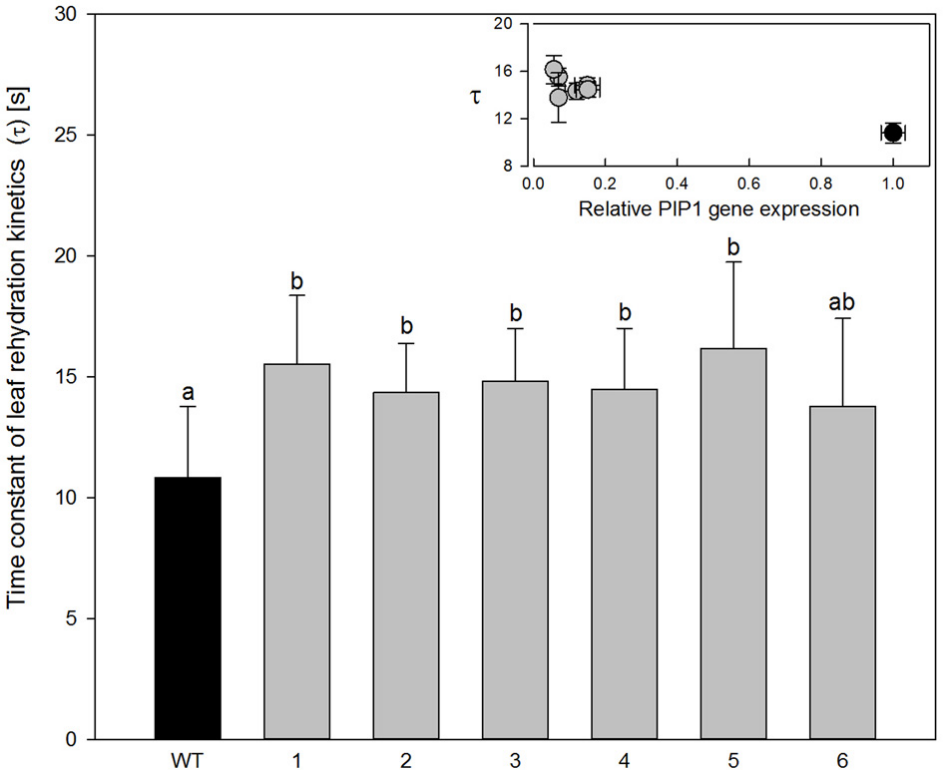
**Time constant of leaf rehydration kinetics for wild-type and transgenic lines**. Data are means of 6–12 leaves for each line; the error bars represent SD. There were significant differences between wild-type and transgenic poplars at *P* < 0.05 according to variance analysis. Letters denote homogeneous groups based on Fisher LSD method corrected for the multiple comparisons. Inset figure; time constant values were plotted for *PIP1* relative expression. Bars denote SE. Black circle, wild-type plants; gray circles, transgenic lines.

Analysis of the leaf rehydration kinetics of wild type *Populus tremula x alba* leaves shows a pattern similar to that of leaf rehydration kinetics in other angiosperm trees (Zwieniecki et al., 2007), with two distinct phases: (1) a fast phase that accounts for approximately 50% of water uptake during rehydration and (2) a slow phase accounting for the remaining observed water uptake. The time constant of the fast phase in wild-type plants was determined to be only 10.8 s. Five out of six transgenic lines showed a significant increase in the time constant of the fast phase by an average of 27% to approximately 15 s (Figure 5; ANOVA *p* = 0.0007). Increase of the time constant was relatively small in relation to relative drop in expression level of PIP1 aquaporins (Figure 5 inset) No significant changes in time constant of the second (slow) phase were observed between species (wild-type average was 238 s while average of for all transgenic plants was 245 s).

## Discussion

Leaves play a key role in plant growth and development, and they need to be continuously supplied with water and carbon dioxide in order to fulfill their photosynthetic functions (Heinen et al., 2009). Although the role of aquaporins as water channels is well accepted, and AtPIP1;2 and NtAQP1 have been confirmed to be CO_2_ transport facilitators (Uehlein et al., 2003; Heckwolf et al., 2011), their involvement in facilitating CO_2_ diffusion in woody plants is still under debate. To elucidate the dual role of aquaporins as facilitators of water and CO_2_ exchange in leaves, we adopted a reverse genetics approach to investigate the effect of the RNAi-mediated suppression of *PIP1* genes on leaf hydraulic and photosynthesis characteristics. Since one aquaporin subfamily is composed of multiple genes with overlapping functions [*Populus trichocarpa PIP1* subfamily consists of five different genes; (Secchi et al., 2009; Almeida-Rodriguez et al., 2010)], we decided that a feasible approach to test their role was to down regulate more genes belonging to the same subfamily in order to reduce the potential for substitution of function or expression compensation within the family. We succeeded in generating several transgenic lines with substantial reductions in *PIP1* gene expression levels of which six randomly selected lines were characterized in detail. These lines did not show a significant effect on PIP2 expression as compensation for *PIP1* down-regulation, despite that fact that all testedisoforms belonging to the *PIP1* subfamily were strongly down-regulated. Accordingly, we believe that the observed phenotypic differences between wild type and transgenic plants can be accounted for by the reduced expression of *PIP1* family members, which specify the role of these genes.

Despite a significant reduction in *PIP1* gene expression level, no morphological differences were observed between wild-type and transgenic plants. This result comes with some level of surprise although previous studies show no strong phenotypes for plants with modified expression of one or more specific aquaporin (Siefritz et al., 2002; Flexas et al., 2006; Heckwolf et al., 2011; Perrone et al., 2012). However, our general expectation was that transgenic plants with reductions of *PIP1* expression level would grow slower due to limitations related to CO_2_ uptake and/or would compensate for higher hydraulic resistance with changes in leaf morphology similar to traits associated with drought stress tolerance. Neither of these expectations was met for plants growing in the greenhouse environment, which were characterized by an ample supply of water, nutrients, and light. It seemed that a massive reduction of *PIP1* aquaporin gene expression was irrelevant to general plant performance under no-stress conditions. This would suggest that increasing *PIP1* transcript level is not strictly related to poplar performance but that these genes might play a secondary role or be important only under stress conditions. A similar conclusion was reported by Siefritz et al. (2002) showing that, despite a strong reduced in NtAQP1 gene expression and root hydraulic conductivity, tobacco plants grown under optimal conditions in the greenhouse did not show morphological changes and that changes in expression did not seem to be important for water uptake. However, in a water limited environment antisense plants were not able to maintain turgor and seemed to be less water stress tolerant (Siefritz et al., 2002).

Even with a lack of any obvious morphological differences between wild-type and transgenic plants, we found specific physiological phenotypes consistent with reduced expression of *PIP1* genes. For example, all transgenic lines showed reductions in mesophyll conductance to CO_2_. This g_m_ reduction was correlated with the expression level of *PIP1*, suggesting a causal relationship between *PIP1*s and g_m_. This finding supports the role of PIP1s in the facilitation of diffusion of CO_2_ across membranes. However, unlike in previous reports (Flexas et al., 2006), we did not observe any significant change in leaf photosynthetic capacity or in levels of carbon isotopic discrimination (δ^13^C). This would suggest that limitations imposed by g_m_ to CO_2_ uptake in poplar were not a limiting factor. In fact, measured g_m_ in wild-type plants was high (~14 μmol m^−2^s^−1^Pa^−1^) and reductions by even 50% and higher still resulted in levels of g_m_ at the level of high performing coniferous leaves (Bickford et al., 2010; Silim et al., 2010; Zhu et al., 2011). It is, however, imperative to add here that CO_2_ curve fitting method used here to estimate g_m_ is not the most reliable and future research with the inline ^13^C should be performed.

Similarly, we observed changes in the hydraulic properties of leaves that were also consistent with expectations, as reduced expression level resulted in a significant increase of the rehydration time constant for the fast water compartment in leaves. Although fast phase in both *wt* and transgenic plants was characterized by one of the fastest time constants among angiosperm species measured using this technique, the relative significance is related to change of the constant that reflects an increase of leaf hydraulic resistance directly involved in the transpiration path (Zwieniecki et al., 2007). As leaf hydraulic resistance was shown to correlate with the stomatal conductance and maximum photosynthetic capacity of leaves (Brodribb et al., 2005), we expected to observe some effect of increased leaf hydraulic resistance on photosynthesis in transgenic lines. However, this was not the case. Neither maximum g*_s_*, nor maximum photosynthetic capacity was affected, and based on δ^13^C results there was no limit in CO_2_ supply that could reflect some difficulty in access to water. However we observed a small increase of δ^15^N in the leaf tissue. This increase could potentially reflect a higher availability of NADPH for N assimilation when competition from CO_2_ is reduced [i.e., lower concentration of CO_2_ in chloroplasts; (Bloom et al., 2010)]. However, only one line showed a significant increase of δ^15^N and ANOVA analysis did support any general conclusion regarding *PIP1* role in change of δ^15^N in transgenic plants.

Overall, we found that although *PIP1* genes were shown to facilitate CO_2_ uptake and contribute to plant hydraulic conductance; under no water stress and under controlled greenhouse conditions they do not have a critical role for the well-being of poplar trees. It seemed that a strong reduction in *PIP1* gene expression has some effects on g_m_ but in a range where it has no physiologically meaningful effect on plant performance despite the fact that reduction of g_m_ should be most relevant under well watered conditions when stomata resistance is at its minimum (i.e., relative contribution of g_m_ to CO_2_ flux is biggest since g_s_ is at its maximum). The same is true for the hydraulic path in the leaf where changes in leaf hydraulic resistance did not produce any detrimental effect on leaf physiology.

Thus, we can conclude that although *PIP1* genes were contributing to the mass transfer of water and CO_2_ in *Populus tremula x alba* leaves, their down-regulation did not dramatically impair the physiological needs of this fast growing tree cultivated under a lack of stress. However, it is possible that PIP1 aquaporins are relevant in the context of water stress via their role in the maintenance of plant water transport capacity (Secchi and Zwieniecki, 2010).

## Materials and methods

### Plant genotypes and construct development

Hybrid white poplar *Populus tremula x Populus Alba* (INRA-France 717-1B4) was chosen for all experimental procedures. An RNAi construct was used to down-regulate *PIP1* expression in poplar and was created in the pHannibal vector (Wesley et al., 2001). A PIP1 cDNA fragment with introduced XhoI and KpnI restriction enzyme sites was amplified by PCR with oligonucleotides designed on conserved *PIP1* domains (listed in Supplemental Table 1) to facilitate directional cloning into the pHannibal vector. Primers were designed in order to down regulate more isoforms belonging to the same subfamily. A second set of PCR primers was used to amplify the same cDNA sequences but with BamHI and ClaI restriction sites to clone the same sequence in the reverse direction into the pHannibal vector. The final pHannibal vector containing both *PIP1* fragments was digested with NotI enzyme and cloned into NotI-digested pART27 binary vector (Gleave, 1992).

### Plant transformation

The vector harboring the insert was inserted into *Agrobacterium tumefaciens* C58/pMP90 (GV3101) strain through freeze/thaw shock transformation (Holsters et al., 1978). Shoot organogenesis was induced directly on mature leaf explants following the protocol by Meilan and Ma (Meilan and Ma, 2006). Briefly, leaf discs (4 mm in diameter) were incubated in Agrobacterium suspension with slow agitation for 1 h. The inoculated explants were co-cultivated in callus-induction medium (CIM) [MS supplemented with 10 μ M naphthaleneacetic acid (NAA) (Sigma, St. Louis, MO) and 5 μ M N6-(2-isopentenyl)adenine, (2iP) (Sigma)] at 22°C in the dark for 2 days. Explants were then washed five times in sterile deionized water and once in wash solution [1/2-strength MS medium, MS vitamins, 1 μ M NAA, 1 μ M 6- benzylaminopurine (BA; *Phyto*Technology Lab., Shawnee Mission, KS), 1 μ M 2iP, 250 mg/L ascorbic acid, and 400 mg/L timentin]. For selection of transformed calli, explants were transferred to CIM containing 25 mg/l kanamycin and 200 mg/l timentin for 21 days. Shoots were induced by culturing explants on SIM medium [MS containing 0.2 μ M Thidiazuron (TDZ; *Phyto*Technology Lab.), 100 mg/l kanamycin, and 200 mg/l timentin] sub-culturing every 3 week until shoots formed. For shoot elongation, explants were transferred onto SEM medium containing 0.1 μ M BA, 100 mg/l kanamycin, and 200 mg/l timentin. The regenerated shoots were rooted on half-strength MS medium supplemented 0.5 μ M indole- 3-butyric acid (IBA; *Phyto*Technology Lab.) and 25 mg/l kanamycin.

To ensure that transformation events were independent, a single clone per individual explant was selected for further propagation. Plantlets were confirmed as transgenic by PCR using *nptII* primers (Supplemental Table 1). Leaf genomic DNA isolation and PCR amplification were achieved using the Extraction-N-Amp Plant PCR Kits according to the manufacturer's instructions (Sigma, Saint Louis, MO, USA). After confirmation of transgene presence, the single plantlets were micro-propagated by repeated subcultural apical cutting on propagation media (half-strength MS medium with no antibiotics).

### Estimation of aquaporin expression levels in transgenic events

Total RNA was isolated from leaf according to the protocol of Chang, (Chang et al., 1993). Contaminant genomic DNA was removed from the samples by digestion with RNase-free DNase I (Fermentas), following the manufacturer's instructions. cDNA was synthesized using SuperScript II Reverse Transcriptase (Invitrogen) according to the supplier's instructions using oligo(dT)12–18 as a primer (Fermentas). Primers designed on *Populus trichocarpa* aquaporin sequences and previously reported (Secchi et al., 2009, 2011) were used to detect the expression level of PIP1.1; PIP1.3; PIP2.5, PIP2.6, and PIP2.8 genes in leaves of hybrid poplars. The oligonucleotide sequences are listed in Supplemental Table 1. Primers were tested on cDNA of hybrid poplar through PCR with RED Taq DNA Polymerase (Sigma) according to the manufacturer's instructions. The expected products were gel purified, inserted into the pGEM-T Easy vector (Promega) and sequenced using M13 forward and reverse primers. The isolated sequences were aligned with the corresponding sequences deposited on Genome Institute's (JGI) *P. trichocarpa* v.1.1 database, (ID numbers are provided in Supplemental Table 1) showing a nucleotide overall identity bigger than 95%. Gene transcript abundance was quantified with SYBR Green JumpStart Taq Ready Mix (Sigma) on an Eco Real-Time PCR System (Illumina, San Diego, USA). Thermocycler conditions for all real-time analyses were: 95°C for 5 min, followed by 40 cycles of 95°C for 15 s, 60°C for 30 s, and 72°C for 30 s.

Data were analyzed using Eco software (Illumina), and the expression values were normalized to the geometric mean of two housekeeping genes (ubiquitin and actin; Supplemental Table 1). These genes were found to have, for the same poplar species, the highest amplification efficiency and most stable expression across different tissues (Carraro et al., 2012). Real-time PCR was carried out using three biological replicates per transformed line. Two technical replicates were performed for each of the three biological replicates.

### Plant growth and physiological measurements

Two months post-propagation plants (about 6 cm tall) were placed into moist potting mix in a 5.7 × 8.3-cm Rose Pot and transferred to a growth room (25 ± 1°C and 16-h photoperiod) for 3 weeks. After acclimation to ambient conditions, plants were transferred to a greenhouse and moved into 1-gallon pots filled with potting mix. Poplars were grown in a greenhouse for 4–6 months. Ambient conditions in the greenhouse were characterized by a temperature maintained in the range of 17–25°C, and the natural daylight was supplemented with light from metal halogen lamps (500–600 μmol photons m^−2^ s^−1^) to maintain a 12/12-h light/dark cycle. Plants were approximately 1.5 m tall at the onset of the experiments.

Six transgenic lines plus *wt* plants were chosen for analysis, each line contained from three to eight plants. Changes in chlorophyll concentration during the leaf maturation process was measured using SPAD meter (SPAD 502 Plus Chlorophyll Meter, Spectrum, Plainfield, IL, USA) on five consecutive leaves with the youngest being approximately 1 cm long. Measurements were repeated twice weekly for 8 weeks till no further increase in chlorophyll concentration was observed (SOM Graph). At that time leaves were fully expanded.

One of these mature leaves was then used for determination of gas exchange parameters. Measurements were made using two LI-COR6400 gas exchange systems, one equipped with the fluorescence chamber (LI-COR, Lincoln, NE, USA). Before measurements, the leaf was acclimated in the gas exchange system at 400 μmol mol^−1^ reference CO_2_ until stomata conductance was stable; this step took about 20 min. Light curves were measured in order to establish the appropriate photosynthetic photon flux density (PPFD) for use in CO_2_ curve responses, 70% of saturating light i.e., 400 μmol m^−2^ s^−1^ as suggested by Gu et al. (2010). General measurement conditions were: PPFD, 400 μmol m^−2^ s^−1^ with 10% blue light component; leaf temperature, 25°C; flow, 200 μmol s^−1^. For the response curves, CO_2_ was reduced from 400 to 40 μmol mol^−1^ in decrements of 40 μmol mol^−1^. After a 9 min re-acclimation at 400 μmol mol^−1^ CO_2_ was increased to 640 (increases of 40 μmol mol^−1^), and then to 700, 800, 900, 1000–2000. Gas exchange was measured at each CO_2_ concentration after the cuvette CO_2_ concentration was stable for 240 s. All measurements were empirically corrected for leaks by evaluating dry poplar leaves under measurement conditions. Standard chlorophyll fluorescence measurement (quantum efficiency of PSII) was made on half of the leaves after recording each gas exchange variable. The fluorescence data were used to better estimate the limiting factors of the curve, and the parameters chosen to fit it were then used for fitting the curve with data collected from gas exchange system (All CO_2_ curve responses are available in SOM materials).

Collected data were then used to estimate A/C*_i_* curve parameters using a previously published fitting procedure (Sharkey et al., 2007). Leaves used for CO_2_ responses were then collected and cut in two along the mid-vein so all leaf parts (tip and base) was included in further analysis; half the leaf was immediately frozen in liquid nitrogen and kept at −80°C until molecular analyses were performed, the other half was dried in an oven and used for isotope analyses.

### Rehydration kinetics

Leaf rehydration kinetic analysis followed the procedure described in an earlier study (Zwieniecki et al., 2007). The “reverse Polish guillotine” was redesigned such that it allowed cutting of a leaf directly off the tree while simultaneously connecting its petiole to water-filled tube in one single motion. Only mature, fully expanded leaves were selected for rehydration measurements. Trees were well-watered with an average water potential below −0.5 MPa. A small portion of the target leaf petiole was and secured in the guillotine holder using foam. A layer of high vacuum silicon grease (Dow Corning, Midland, MI, USA) was added to the petiole to help form a seal with the o-ring. Once system was ready for the cut, water was allowed to flow from the tube such that petiole cut was made in wet conditions. After the entire setup was prepared, the guillotine was deployed such that the leaf was severed from the branch and simultaneously connected to a tube linked to a water reservoir seated on an analytic balance (Sartorius 0.01 mg, Gottingen, Germany). Immediately after the cut, the leaf was placed under water at 20°C. Water uptake was recorded at 1 s intervals for ~1000 [s]. 4–12 leaves per transgenic line were measured for a total of 74 leaves. Leaves were illuminated with 30 μmol m^−2^ s^−1^ PPFD throughout the measurement period, a rate sufficient to saturate any light-induced changes in leaf hydraulic conductance using this measurement technique (Rockwell et al., 2011). After accounting for the evaporation from the measurement system itself, a linear uptake was still observed in all leaves. This residual uptake, which was due to capillary infiltration into leaf intercellular spaces, was also accounted for before further analysis by subtracting it from the generated curve (Zwieniecki et al., 2007).

The weight–time function of water flow from the balance provides a continuous record of leaf rehydration. Following the analysis of a previous study used a double exponential model:

(1) $$f(t)=a*(1-e^{-1/b\;*\;t})+c*(1-e^{-1/d\;*\;t})$$

to describe water uptake by dehydrated leaves. In this model, parameters *a* and *c* describe the volume of the respective two rehydrating phases and *b*, *d* their corresponding time constants. The phases were previously associated with two potential leaf compartments characterized by two different rates of rehydration (Zwieniecki et al., 2007). SLA of each leaf used to determine rehydration kinetic was calculated from dry weight and leaf area as SLA = leaf area/dry weight.

### Isotopes abundance analysis

Isotope analysis was performed in the UC Davis isotope facility. Samples were selected from mid part of the leaf with attention to avoid collecting low order veins from several mature leaves used in the photosynthesis measurements. Samples were then dried at 65°C for several days. Dry material was prepared following online protocol provided by the UC Davis isotope facility (http://stableisotopefacility.ucdavis.edu/13cand15n.html). Briefly leaf tissue was grounded to powder using pestle and mortar. 3–4 mg of material was weighed and packed in aluminum caps and sent for concentration analysis of ^12^C, ^13^C, and ^14^N and ^15^N.

### Statistical analysis

All statistical analysis was performed using Statistica (SoftStat, Inc.).

### Conflict of interest statement

The authors declare that the research was conducted in the absence of any commercial or financial relationships that could be construed as a potential conflict of interest.

## Supplementary material

Supplemental File 1 **Raw data used for the CO_2_ curve-fitting method in the format used by Sharkey et al. (2007)**.

Supplemental Figure 1 **Temporal changes of chlorophyll content in wild-type (black circles and line) and in transgenic plants**. Measurements were repeated in the greenhouse twice weekly for 8 weeks till no further increase in chlorophyll concentration was observed. Data were collected on five consecutive leaves; here we reported values from the youngest leaf being approximately 1 cm long **(A)** and the oldest one **(B)**. Data are means of all available plants from transgenic lines, the error bars represent *SD*.

Supplemental Table 1 **Sequences of primers used for quantitative real time PCR**.
