# Supplementary material for: The physiological response of Populus tremula x alba leaves to the down-regulation of PIP1 aquaporin gene expression under no water stress
Source: Front Plant Sci. 2013 Dec 13;4:507. doi: 10.3389/fpls.2013.00507 (PMC3861612; doi:10.3389/fpls.2013.00507)
Supplement: Supplemental File 1 — Raw data used for the CO2 curve-fitting method in the format used by Sharkey et al. (2007). [file DataSheet1.ZIP › 58650_Secchi_Supplemental Figure 1.PDF]

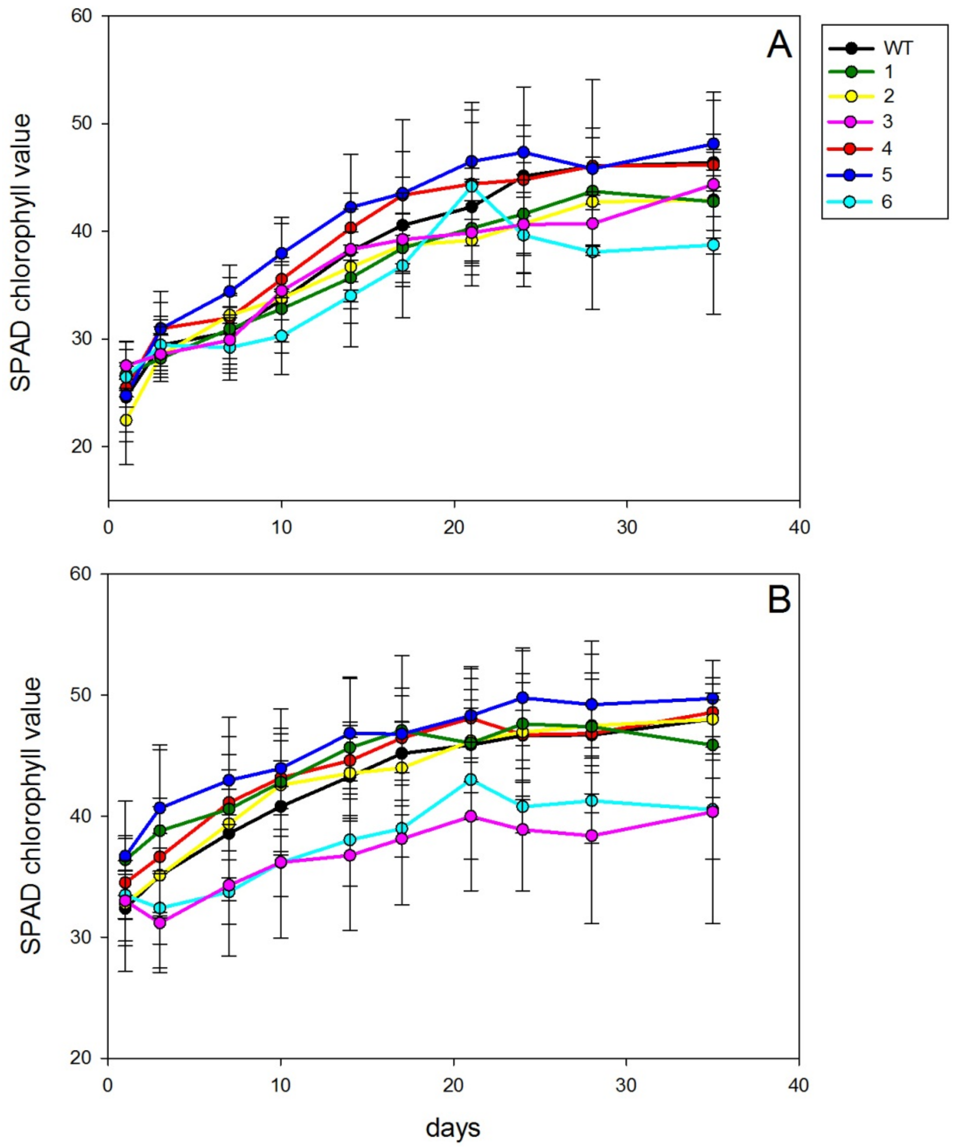

Supplemental Figure 1.

Temporal changes of chlorophyll content in wild-type (black circles and line) and in transgenic plants. Measurements were repeated in the greenhouse twice weekly for 8 weeks till no further increase in chlorophyll concentration was observed. Data were collected on five consecutive leaves; here we reported values from the youngest leaf being approximately 1 cm long (A) and the oldest one (B). Data are means of all available plants from transgenic lines, the error bars represent SD.
