# Supplementary material for: The physiological response of Populus tremula x alba leaves to the down-regulation of PIP1 aquaporin gene expression under no water stress
Source: Front Plant Sci. 2013 Dec 13;4:507. doi: 10.3389/fpls.2013.00507 (PMC3861612; doi:10.3389/fpls.2013.00507)
Supplement: Supplemental File 1 — Raw data used for the CO2 curve-fitting method in the format used by Sharkey et al. (2007). [file DataSheet1.ZIP › 58650_Secchi_Supplemental Table_1.PDF]

Table S1. Sequences of primers used for quantitative real time PCR.

**Gene**	**Primer Sequences 5'-3'**		**Use**
PIP1	For	CCTCGAGGATGGAGGGCAAAGAAGAAGATGTT	1
	Rev	GGGTACCCAATTCCAACCGTTGTGCACTTAGT	
	For	CGGATCCGATGGAGGGCAAAGAAGAA GATGTT	2
	Rev	CCATCGATGGAATTCCAACCGTTGTGCACTTAGT	
nptII	For	GGAGAGGCTATTCGGCTAT	3
	Rev	AACTCGTCAAGAAGGCGATA	
Ubiquitin*	For	CAGCTTGAAGATGGGAGGAC	4
	Rev	CAATGGTGTCTGAGCTCTCG	
Actin*	For	GCAACTGGGATGATATGGAGA	
	Rev	TACGACCACTGGCATACAGG	
PIP2.5**	For	AAGACAAGGCATGGGATGAC	
	Rev	AAGGACCCAAGGGCTTTAAC	
PIP2.6**	For	ATTGGGCGCTGAGATTATTG	
	Rev	CAAATCCAATTGGGAGAGGA	
PIP2.8*	For	CCTTGCGCTCTAAGAACCAC	
	Rev	CAAGCCCAGTTTGTTCCATT	
PIP1.1*	For	CAAGCCCAGTTTGTTCCATT	
	Rev	CAGCCAAACCCCTCAAACTA	
PIP1.3*	For	GTGATGGAGGGCAAAGAAGA	
	Rev	ACAAGAAGGTGGCCATGAAC	

1: primers containing *XhoI and KpnI* restriction sites used for cloning *PIP1* gene into the pHannibal vector

2: primers containing *BamHI* and *ClaI* restriction sites used for cloning *PIP1* gene into the pHannibal vector

3: primers used to detect transgene presence in the genome of transformed plants

4: primers used in Real-Time-PCR to determine transcript abundance

* : primers previously listed by Secchi et al., 2009.

** : primers previously listed by Secchi et al., 2011.

Accession number (ID): ubiquitin, 829282; actin, 746505; PIP2.5, 826419; PIP2.6, 563742; PIP2.8, 836572; PIP1.1, 656216; PIP1.3, 724520.
